# Data on morphometric analysis of the pancreatic islets from C57BL/6 and BALB/c mice

**DOI:** 10.1016/j.dib.2016.07.030

**Published:** 2016-07-20

**Authors:** Thiago Aparecido da Silva, Robertha Mariana Lemes, Carlo Jose Freire Oliveira, Aline da Silva Almeida, Javier Emílio Lazo Chica

**Affiliations:** a Departamento de Biologia Celular e Molecular e Bioagentes Patogênicos da Universidade de São Paulo, Ribeirão Preto, SP, Brazil; b Labotatório de Microbiologia Celular, Instituto Oswaldo Cruz, Rio de Janeiro, RJ, Brazil; c Departamento de Microbiologia, Imunologia e Parasitologia - Universidade Federal do Triângulo Mineiro, Uberaba, MG, Brazil; d Curso de Pós-Graduação em Ciências da Saúde da Universidade Federal do Triângulo Mineiro, Uberaba, MG, Brazil

**Keywords:** Pancreatic islets, Morphometry, BALB/c and C57BL/6 mice

## Abstract

The endocrine portion of the pancreas, which is characterized by pancreatic islets, has been widely investigated among different species. The BALB/c and C57BL/6 mice are extensively used in experimental research, and the morphometric differences in the pancreatic islets of these animals have not been evaluated so far. Thus, our data have a comparative perspective related to the morphometric analysis of area, diameters, circularity, and density of pancreatic islets from BALB/c and C57BL/6 mice. The data presented here are focused to evaluate the differences in morphology of pancreatic islets of two common laboratory mouse strains.

**Specifications Table**

**Table:** 

Subject area	Biology
More specific subject area	Morphometric
Type of data	Graph
How data was acquired	The images of the histological sections of pancreas were obtained using a light microscope light fitted with a digital camera (Evolution MP 5.0; Media Cibernetic Inc., USA) and the Image Pro Plus software (Media Cibernetic Inc., USA). These images were analyzed by ImageJ software to measure the area, major diameter, minor diameter, circularity, and density of pancreatic islets.
Data format	Analyzed and graphed
Experimental factors	The pancreases was dehydrated in ethanol, diaphonized in xylene, and embedded in paraffin. The histological sections were cut at a thickness of 7 μm and were stained with hematoxylin-eosin (H&E).
Experimental features	Quantification of the area, major diameter, minor diameter, circularity, and density of pancreatic islets by morphometric analysis.
Data source location	Federal University of the Triângulo Mineiro, Brazil
Data accessibility	Data is within this article.

**Value of the data**

• The description of the differences in pancreatic islets between BALB/c and C57BL/6 mice favors new perspectives on work with pancreatic experimental models [1], [2], [3], [4].
• The data demonstrate the morphometric analysis of the pancreatic islets from mice using ImageJ and the DeHoff principle, taking this report as application guidelines.
• These data are useful for researchers interested in analyzing the effectiveness and changes to the pancreatic islets in situations such as pancreatitis, diabetes mellitus, and xenotransplantation.

## 1 Data

**Fig. 1:**
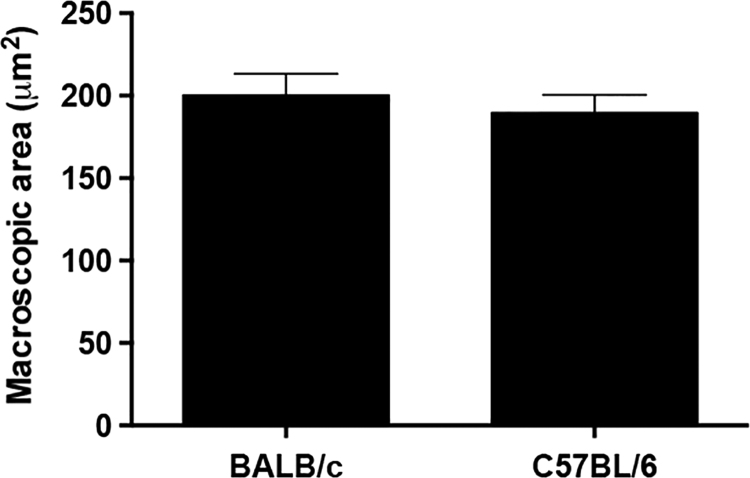
Macroscopic area of C57BL/6 and BALB/c pancreas. The pancreases were removed and imaged. The ImageJ software was used to measure the macroscopic area (μm^2^) of the pancreases. The results have been presented as mean±SEM values.

**Fig. 2:**
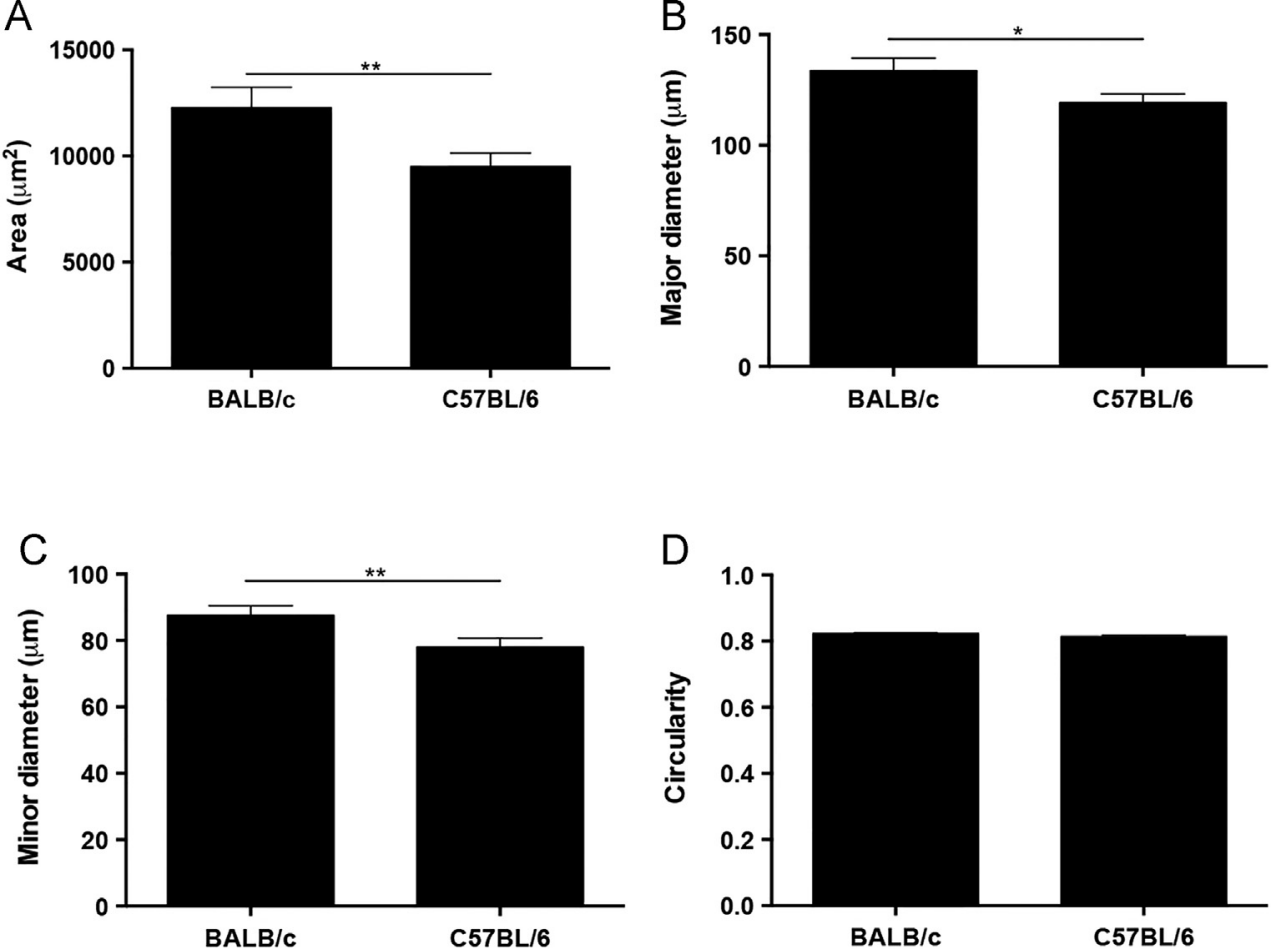
Morphometric analysis of the pancreatic islets from C57BL/6 and BALB/c mice. The ImageJ software was used to measure (A) the area (μm^2^), (B) major diameter (μm), (C) minor diameter (μm), and (D) circularity of the pancreatic islets in both mouse strains. We evaluated 129 pancreatic islets per mouse, with 1556 total measurements taken from 8 histological sections (70 μm between sections) for each mouse strain. The results have been presented as mean±SEM values. *p<0.05 and **p<0.01 were considered statistically significant.

**Fig. 3:**
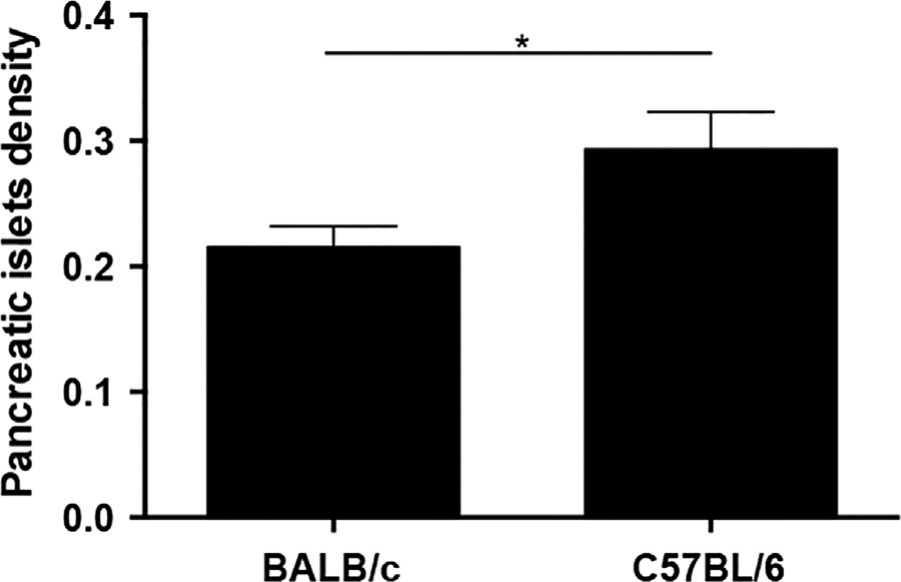
Density of pancreatic islets in BALB/c and C57BL/6 mice. The DeHoff principle was used to calculate the average islet density from two histological sections with an interval of 210 μm. The average density of pancreatic islets was obtained by the formula described in Section 2. The results have been expressed as mean±SEM values. * *p*<0.05 was considered statistically significant.

Fig. 1 shows that the macroscopic pancreatic area of the BALB/c and C57BL/6 mice was not significantly different. The data related to the area and diameter of the pancreatic islets in the C57BL/6 mice were significantly lower than those of BALB/c mice (Fig. 2A–C). In contrast, the circularity of pancreatic islets did not significantly differ between the BALB/c and C57BL/6 mice (Fig. 2D). Fig. 3 shows that the average density of pancreatic islets was significantly higher in the C57BL/6 mice compared to BALB/c mice.

## 2 Experimental design, materials and methods

### 2.1 Materials

6-month-old BALB/c and C57BL/6 male mice were bred and maintained under standard conditions in the animal house of the Department of Cellular Biology in the Federal University of Triangulo Mineiro (UFTM). The animal studies were approved by the Ethical Committee in Animal Research of the Universidade Federal do Triângulo Mineiro (UFTM) (protocol no. 113/2009) and were conducted in accordance with the Ethical Principles in Animal Research adopted by the Brazilian College of Animal Experimentation.

### 2.2 Experimental design

**Fig. 4:**
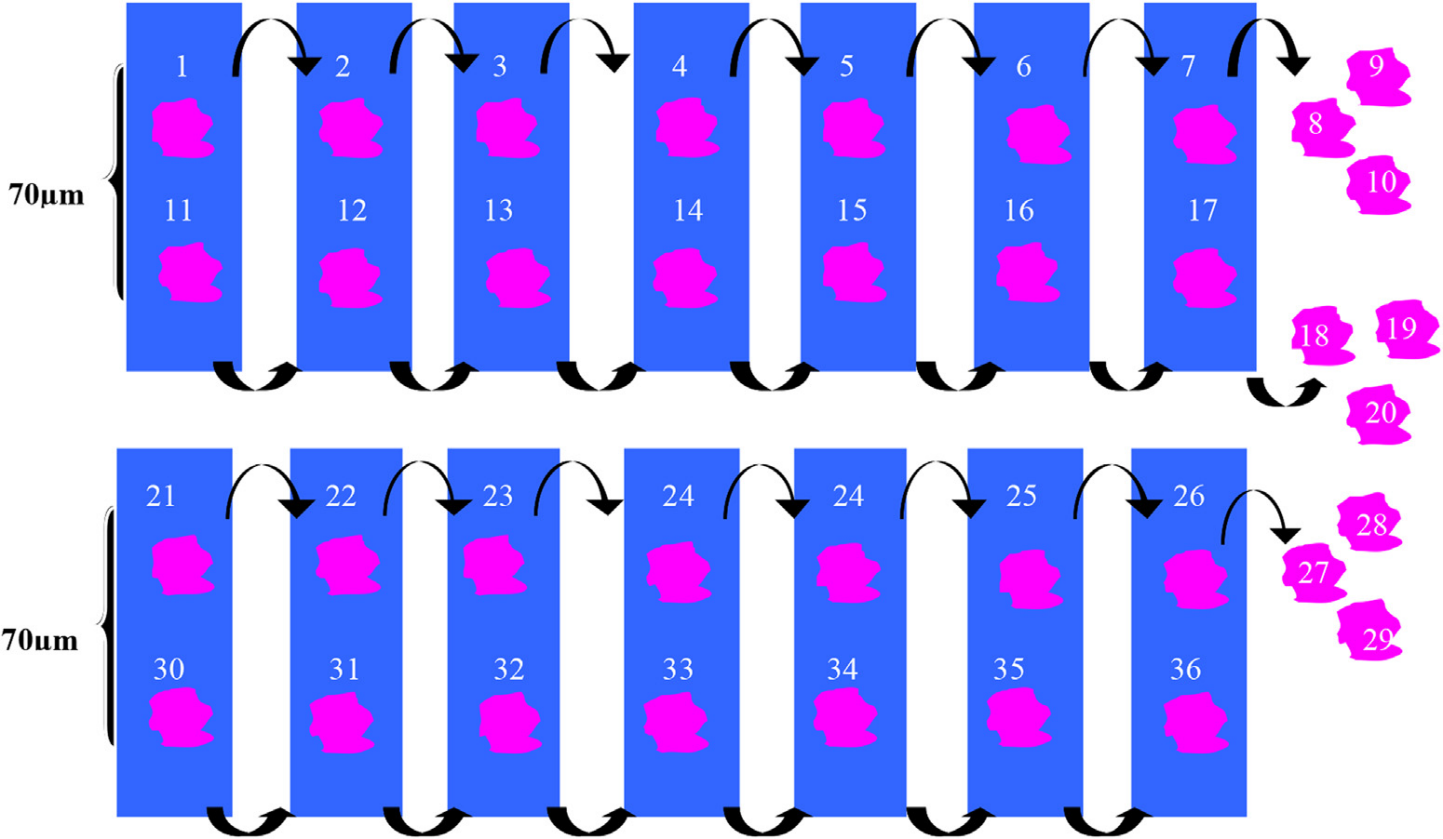
The arrangement of histologic sections of pancreas to morphometric analysis. The histological sections of pancreas (thickness of 7 μm) were distributed on 14 slides with 2 sections/slide. The area (μm^2^), major diameter (μm), minor diameter (μm) and circularity of pancreatic islets (129 pancreatic islets in each mice) were measured in four histological sections with intervals of 70 μm. The number of the pancreatic islets in relation to the pancreas area was determined in two histological sections with intervals of 210 μm in each pancreas.

The pancreas obtained from BALB/c and C57BL/6 mice were photographed to measure the macroscopic area using the ImageJ software. For the histological procedures, the pancreases were fixed in 10% phosphate-buffered formalin for 24 h. The pancreases were dehydrated in ethanol, diaphonized in xylene, and embedded in paraffin. The histological sections were cut at a thickness of 7 μm, and the sections were distributed on 14 slides with 2 sections/slide (Fig. 4). The histological sections were stained with hematoxylin-eosin (H&E). The images of the histological sections were obtained using a light microscope fitted with a digital camera (Evolution MP 5.0; Media Cibernetic Inc., USA) and the Image Pro Plus software (Media Cibernetic Inc., USA). Images of the histological sections were obtained using 10× and 40× objective lenses and were analyzed by ImageJ software.

The pancreatic islets were delimited to measure the area (μm^2^), major diameter (μm), minor diameter (μm) and circularity of pancreatic islets in four histological sections with intervals of 70 μm between each section. The number of pancreatic islets analyzed was determined by the cumulative average [5], with 129 pancreatic islets examined in each pancreas. In total, 1556 pancreatic islets were evaluated in BALB/c or C57BL/6 mice.

The number of the pancreatic islets in relation to the pancreas area was determined by the DeHoff principle [6]. We considered in each pancreas two histological sections with intervals of 210 μm for this analysis. Numerical density (*Nv*) was calculated from the ratio between the number of pancreatic islets in two histological sections (*N*) and the average major diameter of pancreatic islets (*D*), as follows: *Nv*=*N*/*D*.

The results were analyzed using the GraphPad Prism Software, and the values were expressed as mean±standard error means (SEM). Statistical determinations of the difference between means of groups were performed by an unpaired *t*-test with Welch׳s correction. Differences with *p*<0.05 were considered statistically significant.
