# Supplementary material for: Data on morphometric analysis of the pancreatic islets from C57BL/6 and BALB/c mice
Source: Data Brief. 2016 Jul 20;8:1094–8. doi: 10.1016/j.dib.2016.07.030 (PMC4971134; doi:10.1016/j.dib.2016.07.030)
Supplement: Supplementary file 1 — Supplementary material [file mmc1.pdf]

April 22, 2016.

Dear Editor,

We would like to submit enclosed the manuscript "**Data on morphometric analysis of the pancreatic islets from C57BL/6 and BALB/c mice"** by Thiago Aparecido da Silva, Robertha Mariana Lemes, Carlo Jose Freire Oliveira, Aline da Silva Almeida, Javier Emílio Lazo Chica for publication as an original research article in "Data in Brief".

We certify that all authors have seen and approved the final version of the manuscript being submitted. There is no financial interest to report and this work is not under review at any other publication.

Kind regards.

Sincerely yours,

Javier Emilio Lazo Chica, MD, PhD

Please address all correspondence to: Javier Emilio Lazo Chica, Departamento de Biologia Estrutural -Disciplina de Biologia Celular, Universidade Federal do Triângulo Mineiro. Rua Frei Paulino, nº30, 38025-180, Uberaba, MG, Brazil. Phone: +55 34 3318-5626; E-mail: jlazo@dcb.uftm.edu.br; lazo@mednet.com.br
